# Mottling reduction in the early phases of profound septic shock

**DOI:** 10.1186/s13054-019-2558-7

**Published:** 2019-08-02

**Authors:** Daniel Taylor, Duncan Wyncoll

**Affiliations:** Department of Critical Care, St Thomas’ Hospital, Westminster Bridge Rd., Lambeth, London, SE1 7EH UK

We read with great interest the study by Dumas et al. reporting the prognostic value of skin mottling on mortality in a cohort of patients with septic shock [1]. We congratulate the authors on emphasising the utility of this simple bedside clinical tool. In this single-centre observational study, 14-day and ICU mortality increased with the degree of mottling present 6 h after initial resuscitation. A decrease in mottling score during resuscitation was associated with a significant mortality benefit after adjustment for the SOFA score. Traditional resuscitation methods used were in keeping with international sepsis guidelines, the mainstay of which are fluid resuscitation, vasopressors and inotropes.

In those patients with mottling and refractory septic shock, one of the fundamental goals should be to reduce the dose of vasopressor to the minimum necessary. To achieve this, we have adopted the use of femoral arterial lines in such cases; the difference in mean arterial pressure (MAP) between femoral and radial arterial lines may be marked owing to profound peripheral vasoconstriction. Additionally, lowering the target MAP to 55–60 mmHg (away from the Surviving Sepsis initial target of 65 mmHg), employing early use of hydrocortisone and avoiding excessive use of sedation are pragmatic steps to assist vasopressor reduction. Selective use of inodilators, such as levosimendan, may also benefit a cohort of patients with moderate to severely impaired left ventricular function in the setting of septic cardiomyopathy [2].

It is also our institutional practice to commence a low-dose epoprostenol infusion (0.5–10 ng/kg/min) to improve microcirculatory flow. Our experience is that this is an effective way of reducing the mottling score. Intravenous prostacyclin has been demonstrated to increase oxygen delivery in critically ill patients [3] and reverse vasopressor induced peripheral limb ischaemia [4]. Wider adoption of this strategy may be limited by concerns regarding exacerbating hypotension; however, it is our observation that this is avoidable by slow, gradual up-titration of the drug. End points of titration we use are reversal of peripheral mottling and improvement in skin temperature. Low-dose nitroglycerin (0.5–1 mg/h) may have similar effects and contribute to reduced mottling [5].

Management of refractory septic shock is extremely challenging and we must continue to explore alternative methods to improve end-organ perfusion. Whether the aforementioned methods translate into a mortality or limb-saving benefit is unclear and is an area in much need of further study.

## Data Availability

Not applicable.

## Abbreviations

ICU: Intensive care unit
MAP: Mean arterial pressure
SOFA: Sequential Organ Failure Assessment
